# Integrating Biomarkers and Hemodynamics for 1-Year Mortality Risk Stratification in Transcatheter Tricuspid Intervention

**DOI:** 10.1016/j.cjco.2026.03.006

**Published:** 2026-03-27

**Authors:** Christoph Edlinger, Johannes Schlegl, Marwin Bannehr, Michael Lichtenauer, Tanja Kücken, Alexander Krutz, Vera Paar, Michael Neuß, Anja Haase-Fielitz, Christian Butter

**Affiliations:** a Department of Cardiology, Heart Center Brandenburg, Brandenburg Medical School Theodor Fontane (MHB), Bernau/Berlin, Germany; b Faculty of Health Sciences (FGW), Joint Faculty of the University of Potsdam, Brandenburg Medical School Theodor Fontane (MHB) and Brandenburg University of Technology Cottbus-Senftenberg, Cottbus, Germany; c Clinic of Internal Medicine II, Department of Cardiology, Paracelsus Medical University of Salzburg, Salzburg, Austria; d Institute of Social Medicine and Health System Research, Otto von Guericke University Magdeburg, Magdeburg, Germany

**Keywords:** tricuspid regurgitation, transcatheter valve intervention, risk stratification, biomarkers, hemodynamics

## Abstract

**Background:**

Transcatheter tricuspid interventions for severe tricuspid regurgitation are being used increasingly, yet many patients present at an advanced stage, when clinical benefit is limited. The conventional biomarker N-terminal pro-B-type natriuretic peptide (NT-proBNP) often fails to reflect systemic organ injury or right-sided hemodynamics. We hypothesized that systemic biomarkers combined with invasive haemodynamic data improve risk stratification for 1-year mortality after transcatheter tricuspid regurgitation intervention.

**Methods:**

In this prospective, single-centre cohort, preprocedural blood samples for biomarker assessment (growth differentiation factor-15 [GDF-15], soluble urokinase plasminogen activator receptor [suPAR], heart-type fatty acid–binding protein [H-FABP], soluble suppression of tumorigenicity-2 [sST2}, NT-proBNP) and right atrial (RA) pressure were obtained in patients undergoing transcatheter tricuspid repair or valve implantation. Incremental prognostic value was assessed by adding RA pressure to biomarker-based models. Predictive performance for 1-year all-cause mortality was evaluated using receiver operating characteristic analyses. Optimal cutoffs were derived by Youden’s index, and survival was compared using Kaplan–Meier analysis and log-rank testing.

**Results:**

Among 100 patients (mean age, 80.7 ± 6.7 years; 48% male), 22 (22%) died within 1 year. The cohort showed advanced disease and a high comorbidity burden. GDF-15 demonstrated the highest prognostic accuracy (area under the curve [AUC] 0.795, 95% confidence interval [CI] 0.675–0.915; *P* < 0.001), followed by suPAR (AUC 0.746, 95% CI 0.626–0.866; *P* < 0.001). NT-proBNP did not significantly predict mortality. Combining GDF-15 with RA pressure improved discrimination (AUC 0.805). An elevated level of GDF-15 or suPAR was associated with significantly reduced survival.

**Conclusions:**

In patients undergoing transcatheter tricuspid intervention, GDF-15 and suPAR outperform NT-proBNP in predicting 1-year mortality. Integration of RA pressure further refines risk stratification and may aid patient selection and the timing of intervention.

## Résumé

**Contexte:**

La régurgitation tricuspidienne (RT) sévère est de plus en plus souvent traitée par voie transcathéter. Or, de nombreux patients consultent à un stade avancé, lorsque le bienfait clinique de l’intervention est limité. Souvent, le biomarqueur classique, le propeptide natriurétique de type B N-terminal (NT-proBNP), ne rend pas compte des lésions dans l’ensemble du cœur ni de l’hémodynamique cardiaque droite. Nous avons émis l’hypothèse voulant que l’utilisation de biomarqueurs systémiques associée aux données hémodynamiques obtenues à l’aide d’une méthode invasive améliore la stratification du risque de mortalité à 1 an après une intervention transcathéter pour une RT.

**Méthodologie:**

Dans le cadre de cette étude de cohorte prospective menée dans un seul centre, nous avons réalisé des prélèvements sanguins pour évaluer les biomarqueurs (GDF-15, suPAR, H-FABP, sST2, NT-proBNP) et mesuré la pression auriculaire droite (PAD) chez des patients sur le point de subir une réparation ou une implantation transcathéter de la valve tricuspide. Nous avons évalué la valeur pronostique différentielle en ajoutant la mesure de la PAD aux modèles basés sur les biomarqueurs. Nous avons évalué la performance pour prédire la mortalité toutes causes confondues à 1 an en analysant la courbe ROC (*Receiver Operating Characteristic*). Nous avons calculé les seuils optimaux à l’aide de l’indice de Youden, et comparé la survie au moyen de l’analyse de Kaplan-Meier et du test logarithmique par rangs.

**Résultats:**

Sur 100 patients (âge moyen de 80,7 ± 6,7 ans, 48 % d’hommes), 22 (22 %) sont décédés dans l’année. Les sujets de la cohorte étaient à un stade avancé de la maladie et présentaient de nombreuses affections concomitantes. Le GDF-15 s’est traduit par la plus grande précision pronostique (aire sous la courbe [ASC] : 0,795; intervalle de confiance [IC] à 95 % : 0,675 à 0,915; *p* < 0,001), suivi du suPAR (ASC : 0,746; IC à 95 % : 0,626 à 0,866; *p* < 0,001). Le NT-proBNP n’a pas permis de prédire la mortalité de manière significative. Combiner le GDF-15 avec la PAD a amélioré la discrimination (ASC : 0,805). Un taux élevé de GDF-15 ou de suPAR a été associé à une survie significativement plus courte.

**Conclusions:**

Chez les patients qui se prêtent à une intervention transcathéter de la valve tricuspide, le GDF-15 et le suPAR surpassent le NT-proBNP pour prédire la mortalité à 1 an. L’intégration de la PAD permet d’affiner la stratification du risque et pourrait faciliter la sélection des patients et la planification de l’intervention.

Severe tricuspid regurgitation (TR) is recognized increasingly as a progressive condition associated with substantial morbidity and excess mortality, particularly in patients with chronic heart failure.^1^, ^2^, ^3^ Its natural course is characterized by worsening venous congestion, right-sided volume overload, and progressive end-organ dysfunction.^3^ Many patients develop symptoms only at an advanced stage, at which point therapeutic options are limited and the potential for clinical benefit is diminished. Although transcatheter tricuspid interventions have expanded the treatment armamentarium for severe TR, appropriate timing and patient selection remain major challenges, as many individuals are referred too late to derive sustained improvement.^4^

Over the past decade, the field of tricuspid valve disease has undergone a remarkable expansion in scientific activity, particularly in interventional and imaging research. A recent bibliometric analysis demonstrated an exponential increase in global publications since 2018, with a marked shift toward catheter-based therapies and advanced imaging techniques.^5^ Despite this rapid procedural innovation, robust prognostic frameworks for optimal patient selection and timing of intervention remain insufficiently defined.

Risk stratification in this context is difficult, in part because performance of traditional markers such as N-terminal pro-B-type natriuretic peptide (NT-proBNP) is suboptimal. NT-proBNP is influenced by renal dysfunction and systemic congestion and shows only limited association with right-sided hemodynamics and TR severity.^6^^,^^7^ Given that renal impairment and systemic congestion are highly prevalent in this population, the NT-proBNP level may underestimate true disease burden and provide insufficient guidance for procedural selection and timing.

Beyond natriuretic peptides, increasing attention has been directed toward biomarkers reflecting systemic inflammation, oxidative stress, and multiorgan dysfunction in heart failure populations.^8^ Growth differentiation factor-15 (GDF-15) reflects systemic inflammation, oxidative stress, and mitochondrial distress and has demonstrated strong prognostic value across a broad range of cardiovascular conditions.^9^ Mechanistically, GDF-15 has been linked to myocardial stress and remodelling processes, including right ventricular strain.^10^ In the context of tricuspid valve disease, recent data suggest incremental prognostic value following transcatheter intervention.^11^ Soluble urokinase plasminogen activator receptor (suPAR) reflects immune activation and chronic inflammation and has been associated with adverse outcomes in heart failure.^12^^,^^13^ Heart-type fatty acid–binding protein (H-FABP) may indicate ongoing myocardial injury^14^^,^^15^, whereas soluble suppression of tumorigenicity-2 (sST2) reflects myocardial remodelling and fibrosis.^16^^,^^17^

Hepatorenal dysfunction assessed by the **M**odel for **E**nd-Stage **L**iver **D**isease E**x**cluding **I**nternational Normalized Ratio (MELD-XI) score, particularly when integrated with invasive hemodynamic parameters, has been shown to enhance early risk stratification after transcatheter tricuspid valve intervention.^18^ These findings support the concept that adverse outcomes after TR intervention are influenced strongly by systemic disease burden and multiorgan dysfunction rather than isolated valvular pathology alone.

Hemodynamic markers may further refine risk stratification. Right atrial (RA) pressure reflects systemic venous congestion and impaired forward flow, cardinal features of advanced right-sided disease, and has been associated with outcomes in patients with severe TR.^19^ However, whether systemic biomarkers provide clinically meaningful incremental information beyond routinely assessed echocardiographic and hemodynamic parameters, or rather reflect primarily an advanced disease stage, remains insufficiently clarified.

An important point to note is that the present study does not aim to compare interventional vs conservative therapy, but rather to identify high-risk phenotypes within a treated cohort undergoing transcatheter TR intervention. By focusing on 1-year mortality, we extend prior short-term biomarker observations toward midterm outcome prediction and explore whether combining systemic biomarkers with invasive hemodynamics improves discrimination in this elderly high-risk population.

Given the limitations of NT-proBNP level in assessing TR, and emerging evidence supporting biomarkers of systemic stress and immune activation, this study aimed to evaluate the prognostic performance of GDF-15, suPAR, H-FABP, and sST2 for 1-year mortality after transcatheter tricuspid intervention, and determine whether incorporation of RA pressure enhances risk stratification beyond biomarker-only approaches.

## Methods

### Study design and population

**Figure 1:**
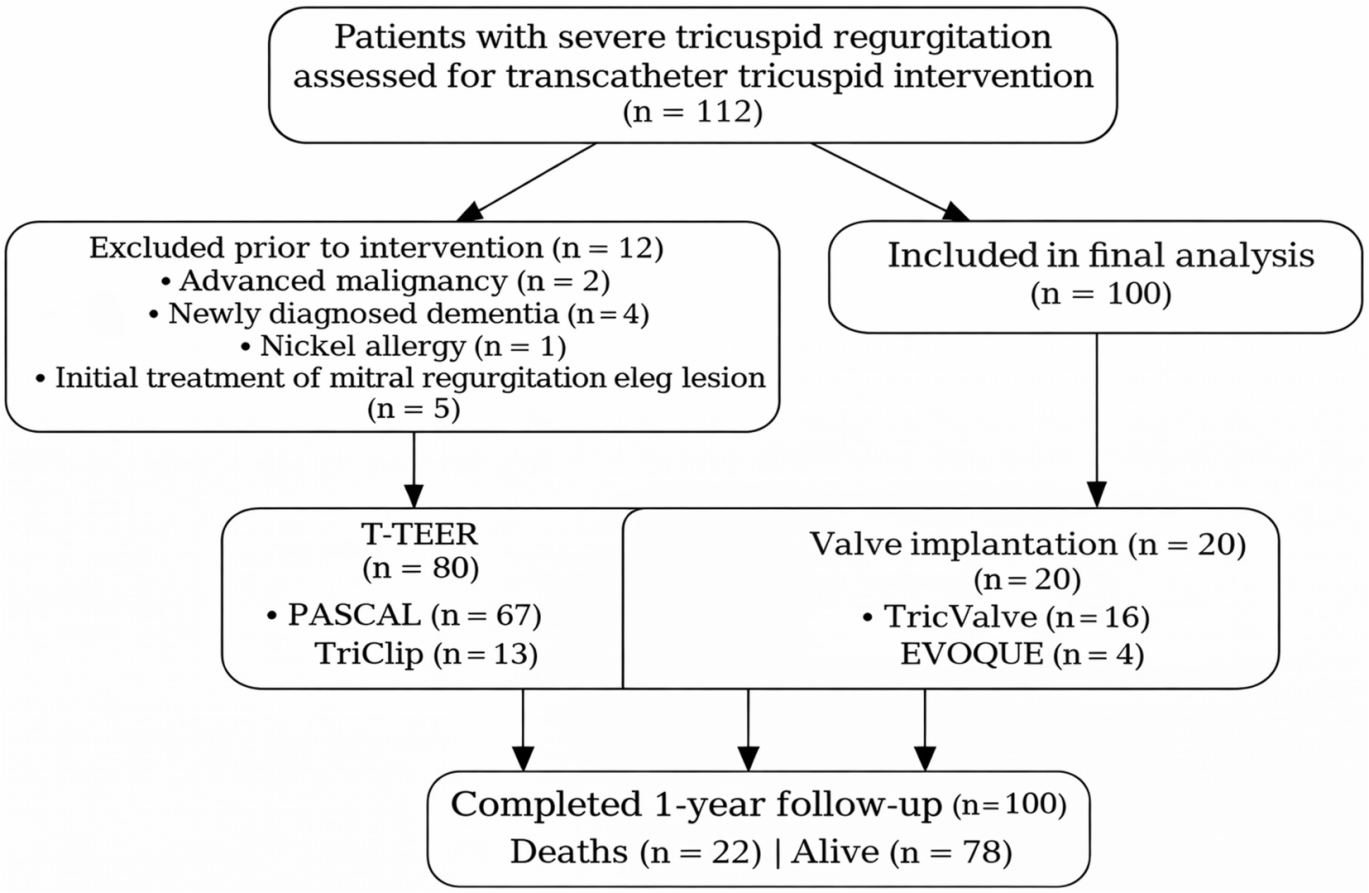
Study flow chart. Flow diagram showing patient screening, inclusion, intervention type, and follow-up to 12 months. EVOQUE System and PASCAL System (Edwards Lifesciences, Irvine, CA); TriClip T-TEER System (Abbott, Santa Clara, CA); TricValve System (P&F Products & Features, Vienna, Austria), T-TEER, transcatheter edge-to-edge repair.

This prospective, single-centre cohort study included consecutive patients undergoing transcatheter tricuspid valve intervention between October 9, 2023 and March 11, 2024. Eligible patients had symptomatic severe TR, despite guideline-directed medical therapy. Exclusion criteria comprised active infection, recent myocardial infarction, advanced malignancy, TR that was no longer severe after medical optimization, technical infeasibility of the intervention, and inability to provide informed consent. Patient selection and study flow are summarized in Figure 1.

Detailed baseline clinical characteristics, including comorbidities (atrial fibrillation, diabetes mellitus, chronic obstructive pulmonary disease, coronary artery disease, chronic kidney disease ≥ IIIb), organ function markers (estimated glomerular filtration rate, MELD and MELD-XI scores), and functional limitation assessed by the 6-minute walk test, were recorded.

All patients underwent comprehensive transthoracic and transesophageal echocardiography at baseline. Left ventricular ejection fraction, mitral regurgitation severity, TR severity according to the Hahn classification, effective regurgitant orifice area, tricuspid regurgitant volume, tricuspid annular plane systolic excursion (TAPSE), and right ventricular longitudinal strain were assessed systematically. Preprocedural right-heart catheterization with comprehensive hemodynamic assessment was performed in all patients, including RA pressure, right ventricular pressure, pulmonary artery pressure, pulmonary capillary wedge pressure, pulmonary vascular resistance, and cardiac output.

The study followed the Declaration of Helsinki and was approved by the Ethics Committee of the Brandenburg Medical School Theodor Fontane (MHB), Germany (approval number: E-01-20190503). All participants provided written informed consent.

### Interventional procedures

All procedures were performed at a tertiary-care centre by a dedicated structural heart team. The choice between transcatheter edge-to-edge repair (T-TEER) and heterotopic caval valve implantation was based on anatomic suitability, right ventricular function, and interdisciplinary heart team consensus. T-TEER was performed using either the Pascal T-TEER System (Edwards Lifesciences, Irvine, CA) or the TriClip T-TEER System (Abbott, Santa Clara, CA).

The Pascal T-TEER System incorporates a central spacer designed to facilitate leaflet coaptation and reduce regurgitation and has demonstrated feasibility and clinical effectiveness in patients with severe TR.^20^ The TriClip T-TEER System, derived from MitraClip technology (Abbott Laboratories, Abbott Park, IL) and specifically adapted for the tricuspid valve, relies on direct leaflet approximation without a spacer.^21^ Both systems have demonstrated procedural safety and feasibility in prospective studies and real-world cohorts.^22^, ^23^, ^24^

In patients unsuitable for T-TEER due to anatomic constraints, heterotopic caval valve implantation was performed using the TricValve System (P&F Products & Features, Vienna, Austria). This approach involves implantation of self-expanding bioprosthetic valves into the superior and inferior vena cava to reduce systemic venous congestion without directly modifying the native tricuspid valve.^25^^,^^26^

In selected patients with severe TR who were unsuitable for leaflet-based repair due to anatomic constraints (eg, large coaptation gaps, extensive tethering, or unfavourable leaflet morphology), transcatheter tricuspid valve replacement was performed using the EVOQUE System (Edwards Lifesciences). The EVOQUE device is a self-expanding bioprosthesis designed for orthotopic implantation in the native tricuspid annulus, with the aim of eliminating regurgitation by valve replacement rather than leaflet approximation. Procedural planning and device selection were guided by multimodality imaging and heart-team consensus, consistent with contemporary clinical evidence for orthotopic transcatheter tricuspid valve replacement in high-risk populations.^27^

## Results

### Study population

A total of 100 consecutive patients with severe TR undergoing transcatheter tricuspid intervention were included. Baseline characteristics are shown in Table 1. The mean age was 80.7 ± 6.7 years, and 48% were male. The comorbidity burden was high, including impaired renal function (mean estimated glomerular filtration rate 42.5 ± 15.6 mL/min per 1.73 m^2^) and elevated MELD and MELD-XI scores.

**Table 1:** Baseline characteristics

Variable	All (N = 100)
Demographics	
Sex, male	48 (48)
Age, Y	80.7 ± 6.7
Body mass index, kg/m^2^	26.9 ± 5.9
Type of intervention	
Edge-to-edge repair	80 (80)
Pascal T-TEER System	67 (67)
TriClip T-TEER System	13 (13)
Heterotopic caval valve implantation (TricValve System)	16 (16)
EVOQUE System	4 (4)
Scores and comorbidities	
MELD score, points	14.02 ± 4.41
MELD XI score, points	14.28 ± 4.18
eGFR, mL/min per 1.73 m^2^	42.47 ± 15.62
Type II diabetes	35 (35)
Coronary artery disease	52 (52)
Atrial fibrillation	97 (97)
Paroxismal	13 (13,4)
Persistent	33 (34)
Permanent	51 (52,6)
COPD	19 (19)
Hypertension	75 (75)
Dyslipidemia	71 (71)
Echocardiographic parameters	
LVEF, %	55 (45–60)
TAPSE, mm	16 (14–18)
Mitral regurgitation	1,0 (1,0–1,5)
RV strain	-23 (-18– -25)
Invasive hemodynamic parameters	
Right atrial pressure, mm Hg	11 (8–15)
Right ventricular pressure, mm Hg	10 (8–14)
Pulmonary artery pressure, mm Hg	26 (21–32)
Pulmonary capillary wedge pressure, mm Hg	17.5 (14–22)
Pulmonary vascular resistance, WU	2.19 (1.46–3.02)
Cardiac output, L/min	4.07 (3.43–4.87)

Values are n (%), mean ± standard deviation, or median (interquartile range). Pascal T-TEER System and EVOQUE System (both from Edwards Lifesciences, Irvine, CA); TriClip T-TEER System (Abbott, Santa Clara, CA). TricValve, System (P&F Products & Features, Vienna, Austria).

COPD, chronic obstructive pulmonary disease; eGFR, estimated glomerular filtration rate; LVEF, left ventricular ejection fraction; MELD, **M**odel for **E**nd-Stage **L**iver **D**isease; MELD-XI, MELD E**x**cluding **I**nternational Normalized Ratio; RV, right ventricular; TAPSE, tricuspid annular plane systolic excursion; WU, Wood units.

T-TEER was performed in 80 patients (80%), using primarily the Pascal T-TEER System (67%), whereas 13% received TriClip. Valve implantation strategies included heterotopic caval valve implantation with TricValve in 16 patients (16%) and orthotopic replacement with the EVOQUE System in 4 patients (4%). Preprocedural right-heart catheterization with assessment of RA pressure was performed in all patients.

### Clinical outcomes

During 1-year follow-up, 22 patients (22%) died. Complete follow-up data were available for all patients. Baseline characteristics stratified by 1-year survival are shown in Table 2.

**Table 2:** Baseline characteristics stratified by 1-year survival

Variable	Survivors (N = 78)	Non-survivors (N = 22)	*P*
Sex, male	39 (50)	9 (43)	0.479
Age, Y	80.6 ± 7.2	81.0 ± 4.6	0.725
Body mass index, kg/m^2^	26.7 ± 5.2	26.6 ± 5.3	0.936
MELD score, points	13.0 (IQR 6.0)	17.0 (IQR 7.0)	0.039
MELD XI score, points	13.5 (IQR 5.0)	17.0 (IQR 9.0)	0.032
eGFR, mL/min per 1.73 m^2^]	41.0 (IQR 15.0)	35.0 (IQR 17.0)	0.069
Left ventricular ejection fraction, %	50.7 ± 9.7	51.6 ± 10.1	0.591
TAPSE, mm	16.2 ± 3.9	15.2 ± 3.5	0.518
Right atrial pressure, mm Hg	11 (IQR 8–14)	14 (IQR 12–20)	0.018
Mitral regurgitation	1 (IQR 1–2)	1 (IQR 1–2)	0.640
Type II diabetes	27 (34.6)	8 (36.4)	0.534
Coronary artery disease	44 (56.4)	8 (36.4)	0.146
Atrial fibrillation	75 (96.2)	22 (100)	0.670
Chronic obstructive pulmonary disease	12 (15.4)	7 (31.8)	0.121
Hypertension	56 (71.8)	19 (86.4)	0.264
Dyslipidemia	56 (71.8)	15 (68.2)	0.793

Values are n (%), or mean ± SD, or median (interquartile range [IQR]), unless otherwise indicated.

Differences between survivors and non-survivors was assessed using the χ^2^, Student *t* test, or Mann-Whitney-U test, as appropriate.

eGFR, estimated glomerular filtration rate; MELD, **M**odel for **E**nd-Stage **L**iver **D**isease; MELD-XI, MELD E**x**cluding **I**nternational Normalized Ratio; TAPSE, tricuspid annular plane systolic excursion.

### Biomarker performance for 1-year mortality

Preprocedural levels of GDF-15 and suPAR were significantly higher in patients who died during the follow-up period, compared with survivors, whereas NT-proBNP level showed substantial overlap between groups. Differences in H-FABP and sST2 levels were less pronounced. Baseline biomarker concentrations are shown in Table 3.

**Table 3:** Biomarker concentrations

Biomarker concentration	All (N = 100)	Survivors (N = 78)	Non-survivors (N = 22)	*P*
GDF-15, pg/mL	1667 (1517)	1546 (1117)	3435 (2787)	< 0.001
suPAR, ng/mL	3879 (1722)	3633 (1600)	4721 (3199)	< 0.001
NT-proBNP, pg/mL	2465 (3026)	2380 (2958)	2662 (3939)	0.160
H-FABP, pg/mL	2043 (1781)	1993 (1212)	3218 (4131)	0.054
sST2, pg/mL	2542 (2094)	2504 (1900)	3278 (3315)	0.055

Data are presented as value (interquartile range), unless otherwise indicated. Median baseline serum/plasma biomarker concentrations for all patients, separated by 1-year survival. Differences between survivors and non-survivors were assessed using the Mann-Whitney-U test.

GDF-15, growth differentiation factor-15; H-FABP, heart-type fatty acid–binding protein; NT-proBNP, N-terminal pro-B-type natriuretic peptide; sST2, soluble suppression of tumorigenicity-2; suPAR, soluble urokinase plasminogen activator receptor.

**Figure 2:**
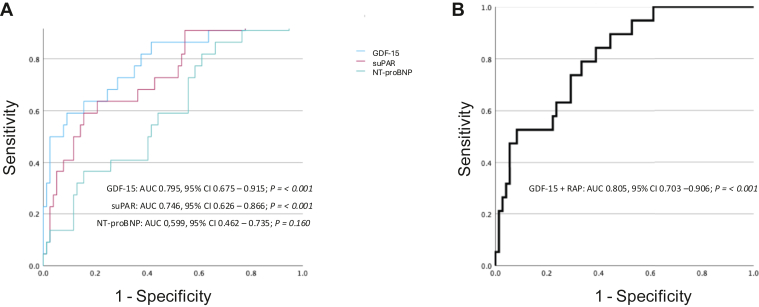
(**A**) Predictive value for mortality: growth differentiation factor-15 (GDF-15) and soluble urokinase plasminogen activator receptor (suPAR) vs N-terminal pro-B-type natriuretic peptide (NT-proBNP). Receiver operating characteristic curves comparing biomarker performance for 1-year all-cause mortality. (**B**) Prognostic performance of GDF-15 when combined with right arterial pressure obtained from pre-interventional right heart catheterization (RAP). Receiver operating characteristic curves for biomarker prediction of 1-year all-cause mortality. AUC, area under the curve; CI, confidence interval; NT-proBNP, N-terminal pro-B-type natriuretic peptide; suPAR, soluble urokinase plasminogen activator receptor.

In receiver operating characteristic (ROC) analysis, GDF-15 demonstrated the highest discriminatory performance for 1-year all-cause mortality (area under the curve [AUC] 0.795, 95% confidence interval [CI] 0.675-0.915; *P* < 0.001), followed by suPAR (AUC 0.746, 95% CI 0.626-0.866; *P* < 0.001). NT-proBNP level did not significantly predict mortality (AUC 0.599, 95% CI 0.462-0.735; *P* = 0.160; Fig. 2A). Prognostic performance of H-FABP (AUC 0.640, 95% CI 0.493-0.787; *P* = 0.046) and sST2 (AUC 0.636, 95% CI 0.496-0.776) was modest and inferior to that of GDF-15 and suPAR.

### Combined biomarker and hemodynamic assessment

RA pressure alone showed moderate predictive ability for mortality. Combining GDF-15 with RA pressure improved prognostic performance, resulting in an AUC of 0.805 (95% CI 0.703-0.906; *P* < 0.001), exceeding the discriminatory capacity of either parameter alone (Fig. 2B).

### Survival analysis and biomarker cutoffs

Using Youden’s Index, optimal cutoff values for mortality were identified at 2400 pg/mL for GDF-15, and 3350 pg/mL for suPAR. Accordingly, 31 patients had GDF-15 levels above the cutoff, and 63 patients had suPAR levels above the cutoff.

**Figure 3:**
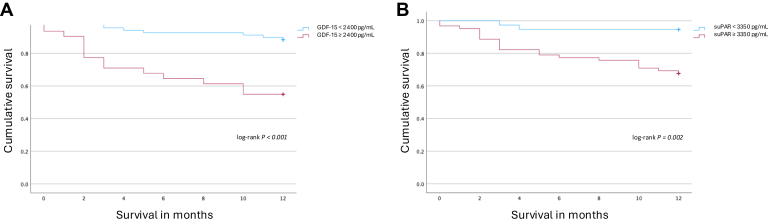
(**A**) Kaplan–Meier curve stratified by growth differentiation factor-15 (GDF-15). Survival curves for 1-year all-cause mortality according to GDF-15 threshold (≥ 2400 pg/mL vs < 2400 pg/mL). (**B**) Kaplan–Meier curve stratified by soluble urokinase plasminogen activator receptor (suPAR). Survival curves for 1-year all-cause mortality according to suPAR threshold (≥ 3350 ng/mL vs < 3350 ng/mL).

Kaplan–Meier analysis demonstrated significantly reduced 1-year survival in patients with GDF-15 ≥ 2400 pg/mL, compared with those below this threshold (log-rank *P* < 0.001; Fig. 3A). Similarly, patients with suPAR ≥ 3350 pg/mL had significantly higher mortality incidence than those with lower levels (log-rank *P* = 0.002; Fig. 3B). Patients with elevations in levels of both biomarkers exhibited the poorest survival. Given the limited number of events (n = 22), multivariable adjustment was not performed, to avoid model overfitting, and survival analyses were restricted to univariable Kaplan–Meier comparisons.

## Discussion

In this prospective, single-centre cohort of patients undergoing transcatheter tricuspid valve intervention, elevated levels of systemic biomarkers were associated with increased 1-year mortality. These findings underscore the persistent heterogeneity of outcomes despite technically successful intervention and reflect the complex interplay between valvular pathology and systemic disease burden.^28^

Although transcatheter therapies have significantly expanded treatment options for patients with severe TR who are not surgical candidates, midterm mortality incidence remains considerable in contemporary multicentre studies and real-world experiences.^22^, ^23^, ^24^ This apparent discrepancy between high procedural success and residual mortality suggests that outcome is not determined by valvular correction alone but rather by the overall stage of right-sided heart failure and systemic involvement.

Our data reinforce the concept that systemic congestion and multiorgan dysfunction are central determinants of prognosis. RA pressure, as a direct marker of venous congestion, has been linked previously to adverse outcomes in severe TR.^19^ In advanced stages of disease, prolonged venous hypertension may lead to progressive hepatic and renal dysfunction, further aggravating clinical instability. The integration of circulating biomarkers with invasive hemodynamics may therefore better reflect global disease severity rather than isolated valvular dysfunction.

An important finding is that biomarkers such as GDF-15 and suPAR reflect inflammatory activation and systemic stress rather than tricuspid valve–specific mechanisms alone.^9^^,^^12^ Their prognostic signal may therefore capture predominantly advanced disease stage, rather than providing pathophysiological specificity for tricuspid valve pathology. This interpretation is supported by the observation that markers of organ dysfunction were more pronounced in non-survivors, indicating that multiorgan involvement substantially contributes to outcome.

Although the combination of GDF-15 and RA pressure numerically improved risk discrimination, compared with individual parameters, this improvement was modest. Given the limited event count and single-centre design, the present findings should be interpreted as hypothesis-generating rather than definitive evidence of incremental prognostic superiority. Larger prospective cohorts are necessary to determine whether biomarker integration meaningfully improves clinical decision-making beyond established clinical and hemodynamic parameters.

From a clinical perspective, these findings may have implications for patient selection and timing of intervention. If adverse outcomes after transcatheter therapy are driven primarily by advanced systemic disease, earlier identification of high-risk phenotypes could become relevant. However, this study was not designed to compare interventional therapy with conservative management, and no conclusions regarding treatment allocation can be drawn. Rather, the objective was to characterize prognostic phenotypes within a treated population undergoing contemporary transcatheter tricuspid valve intervention.

Taken together, our results suggest that outcome after transcatheter tricuspid intervention is strongly influenced by systemic disease burden and multiorgan involvement. Biomarker integration in combination with invasive hemodynamics may refine risk stratification, but prospective validation in larger, multicentre studies is required.

This study has several limitations. It represents a single-centre experience with a limited sample size and event count, which restricts statistical power and precluded multivariable adjustment to avoid overfitting. Biomarker assessment was performed at baseline only, and dynamic changes over time were not evaluated. In addition, unmeasured confounders and residual selection bias inherent to observational designs cannot be excluded. Therefore, the findings should be interpreted as exploratory and hypothesis-generating and require confirmation in larger, prospective, multicentre cohorts.

## Conclusion

In this single-centre cohort of patients undergoing transcatheter tricuspid valve intervention, elevated systemic biomarkers were associated with increased 1-year mortality. These findings suggest that outcomes after contemporary transcatheter therapy are influenced strongly by systemic disease burden and multiorgan dysfunction, rather than isolated valvular pathology alone.

Integration of circulating biomarkers with invasive hemodynamic parameters may improve risk stratification within treated populations. However, given the exploratory design and limited event count, these results should be considered hypothesis-generating and require validation in larger prospective cohorts.
